# Disseminated Alveolar Hydatid Disease Resembling a Metastatic Malignancy: A Diagnostic Challenge—A Report of Two Cases

**DOI:** 10.1155/2014/638375

**Published:** 2014-10-08

**Authors:** Mesut Bulakci, Erdem Yilmaz, Ferhat Cengel, Ahmet Gocmez, Merve Gulbiz Kartal, Emine Goknur Isik, Erhan Celenk, Gulcin Yegen, Artur Salmaslioglu

**Affiliations:** ^1^Department of Radiology, Istanbul Faculty of Medicine, Istanbul University, Millet Caddesi, Capa, 34093 Istanbul, Turkey; ^2^Department of Radiology, Haseki Training and Research Hospital, 34096 Istanbul, Turkey; ^3^Department of Nuclear Medicine, Istanbul Faculty of Medicine, Istanbul University, 34093 Istanbul, Turkey; ^4^Department of Pathology, Istanbul Faculty of Medicine, Istanbul University, 34093 Istanbul, Turkey

## Abstract

Alveolar hydatid disease or alveolar echinococcosis is a disease of the parasite *Echinococcus multilocularis* that is potentially fatal if left untreated. It primarily involves the liver but can be disseminated to other organs like the lungs and the brain by hematogenous route. Multiorgan involvement and the aggressive appearance of lesions make alveolar hydatid disease easy to confuse with a metastatic malignancy. For this reason, histopathological confirmation is essential for definite diagnosis. We present the imaging features of this disease in two patients in order to emphasize that these lesions can be easily misdiagnosed as malignancies.

## 1. Introduction

Echinococcosis, also referred to as hydatid disease, is a general term used to define zoonosis caused by Echinococcus tapeworms, or cestodes. The two main types are cystic echinococcosis, caused by* Echinococcus granulosus* and seen worldwide, and alveolar echinococcosis (AE), caused by* Echinococcus multilocularis* and restricted to the northern hemisphere [1]. Humans are the accidental intermediate host in the life cycle of this genus, usually acquiring the parasite by ingesting egg-contaminated foods. Although the liver is the main organ involved, the lungs and other organs may be affected as well [1, 2].

## 2. Case Report

### 2.1. Case  1

A 40-year-old male patient presented to our hospital with complaints of right upper quadrant pain and weight loss. The patient was in good general condition, with no respiratory distress. Except for mild hepatomegaly, physical examination was completely normal. It was noted in the patient's detailed history that he lived in rural Eastern Anatolia. Hemogram results were normal, with no signs of peripheral eosinophilia.

**Figure 1:**
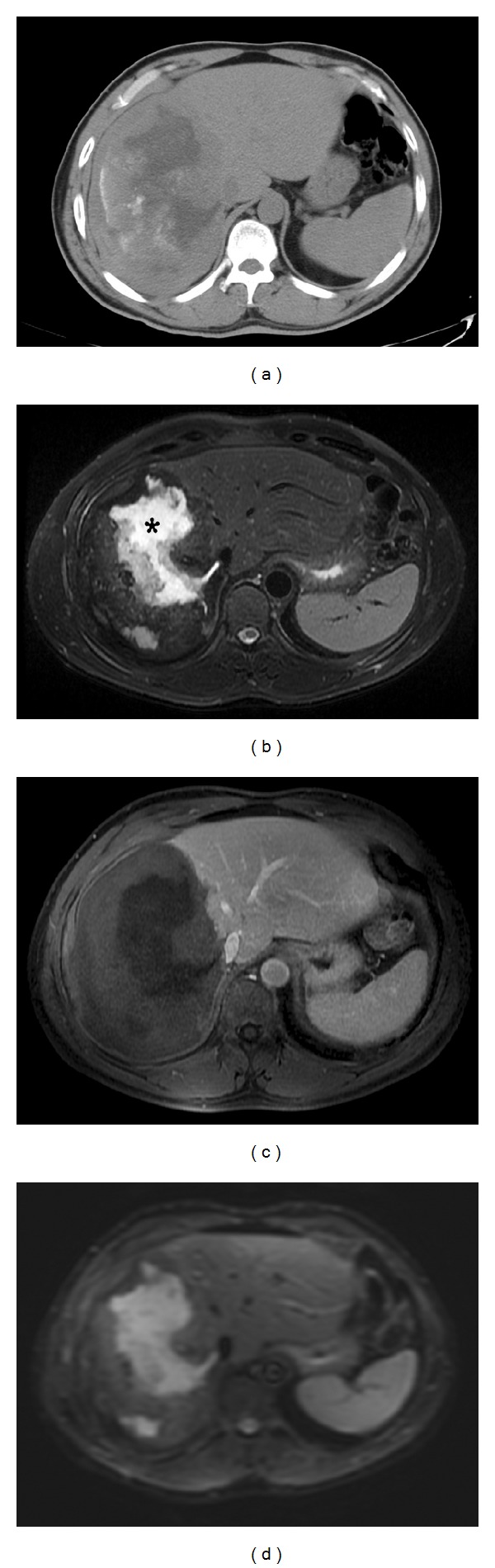
Unenhanced CT scan shows an infiltrating hepatic solid mass that contains multiple cystic-necrotic hypodense areas and hyperattenuating foci of internal calcifications (a). Necrotic areas (asterisk) of the mass are clearly seen in an axial T2-weighted fast recovery fast spin echo (FRFSE) image (b). Gadolinium-enhanced T1-weighted gradient-echo, dynamic 3D-liver acquisition with volume acceleration (LAVA) image depicts no contrast enhancement in the lesion (c). Relative to the surrounding liver parenchyma, the central areas of the mass remain hyperintense on the diffusion-weighted (*b* value = 500 s/mm^2^) spin echo-echo planar imaging (SE-EPI) image (d).

Abdominal ultrasonography (US) revealed a large heterogeneous solid mass lesion containing cystic and calcified areas in the right lobe of the liver. Thoracoabdominal computed tomography (CT) revealed a heterogeneous solid mass lesion measuring 15 × 10 cm that contained multiple hypodense cystic areas and calcified foci (Figure 1(a)). T2-weighted magnetic resonance (MR) images clearly demonstrated extensive central cystic-necrotic areas (Figure 1(b)). Abdominal dynamic contrast enhanced magnetic resonance imaging (MRI) showed no contrast enhancement in the lesion (Figure 1(c)). Diffusion-weighted MR images obtained with *b* values of 500 sec/mm^2^ show signal hyperintensity in the central areas of the mass (Figure 1(d)).

**Figure 2:**
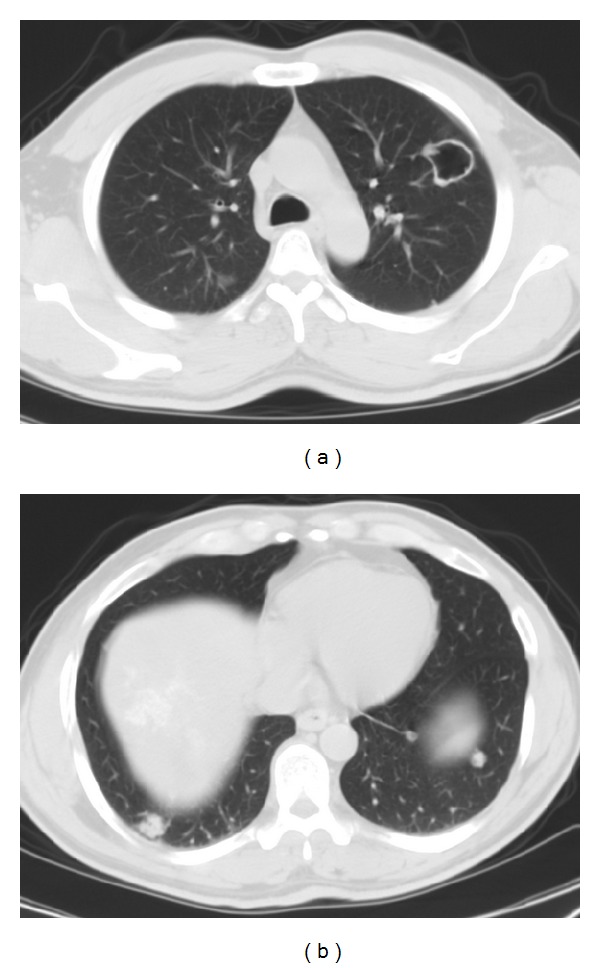
Thorax CT reveals multiple noncalcified nodular lesions in both lungs (a). A cavitary lesion with thick irregular wall is seen in the upper lobe of the left lung (b).

There were also multiple scattered lesions more prominent in the peripheral zones of both lungs (Figure 2). No other clinical or radiological findings were noted. Radiological images were suggestive of a liver malignancy that had metastasized to the lungs. US-guided core needle biopsy was performed for histopathological analysis. Pathologic examination revealed germinative membranous structures of* Echinococcus multilocularis* in the liver parenchyma that were consistent with hepatic alveolar echinococcosis (Figure 6(a)).

### 2.2. Case  2

**Figure 3:**
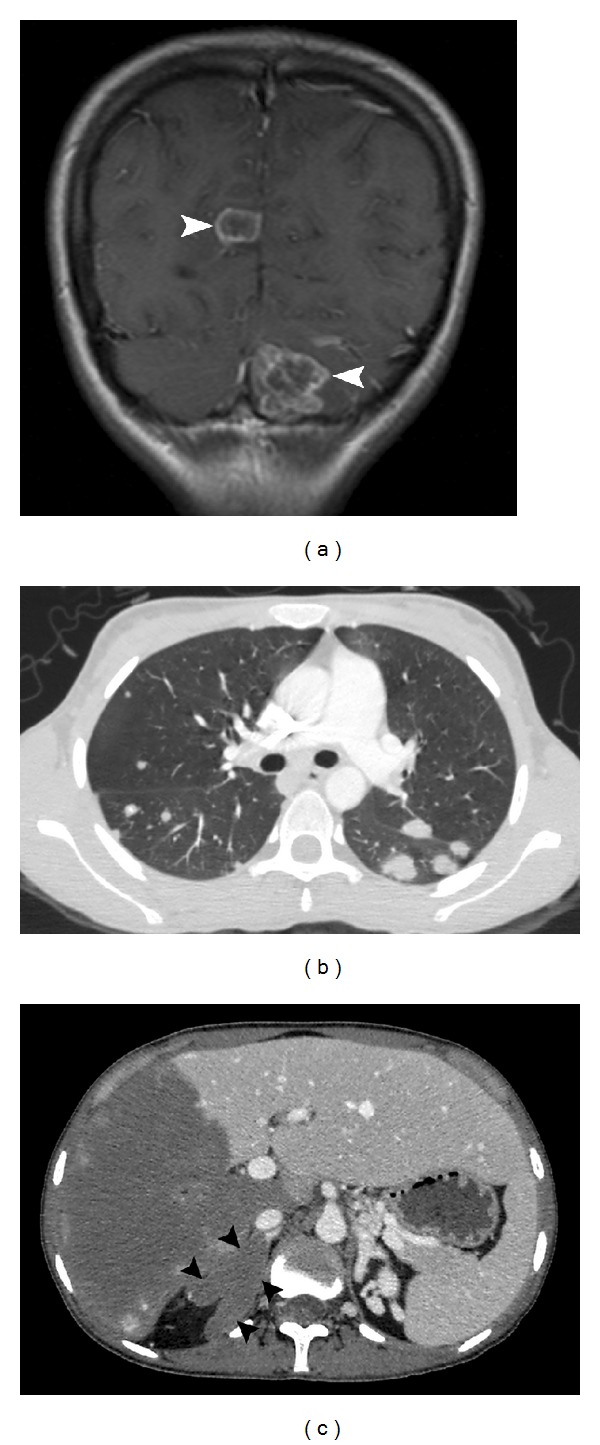
Postcontrast coronal T1-weighted spin echo (SE) image (a) showing irregular peripheral enhancement at the lesions (arrowheads). Chest CT image (b) demonstrates bilateral pulmonary involvement of alveolar hydatid disease. Diffuse right adrenal gland enlargement (arrowheads) is present on contrasted CT (c) compared to the left gland.

A 21-year-old female patient was referred to our hospital with complaints of headache, nausea, and vomiting that had persisted for the past three months. It was noted during history taking that the patient lived in rural Eastern Anatolia. Contrast enhanced cranial MRI showed two lesions with irregular annular contrast enhancement, one measuring 15 × 12 mm at the right occipital lobe and a 25 × 24 mm one in the left cerebellar hemisphere (Figure 3(a)). Lesions were isointense with cerebral gray matter on T1-weighted images and showed heterogeneous low signal intensity on T2-weighted images. There was increased signal intensity around the lesions consistent with edema on T2-weighted and FLAIR images.

**Figure 4:**
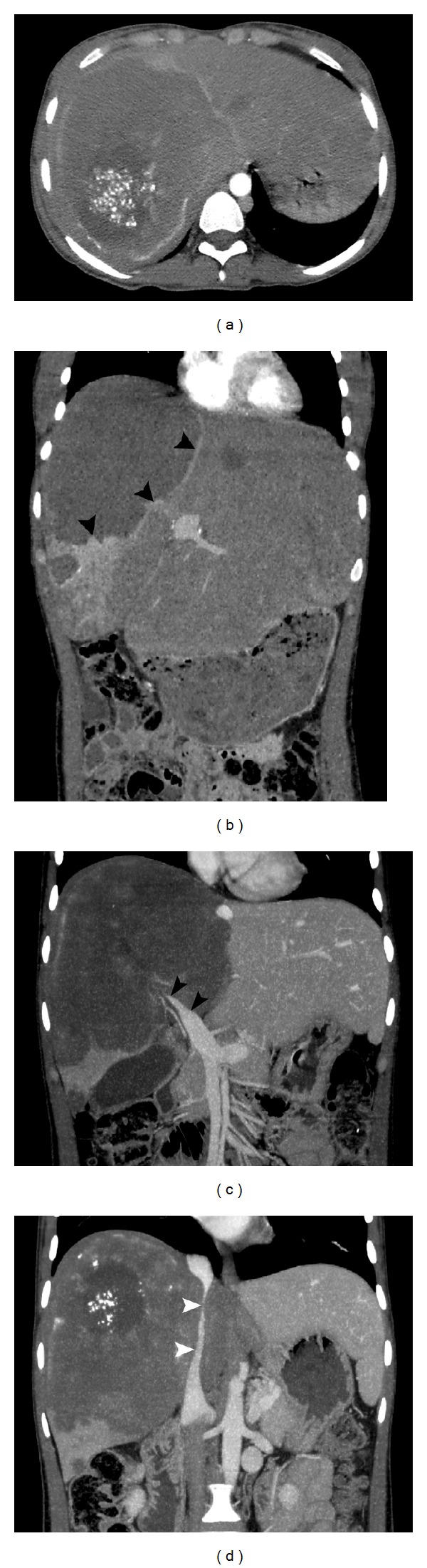
Axial enhanced abdominal CT image (a) demonstrates an infiltrative tumor-like hepatic lesion with irregular margins and heterogeneous contents, including amorphous calcifications. The fibroinflammatory tissue surrounding the mass (arrowheads) exhibits mild contrast enhancement in a delayed phase coronal reformatted CT image (b). Right portal vein branch thrombosis (arrowheads) is shown in acoronal contrast-enhanced maximum intensity projection (MIP) image (c). Another coronal MIP image (d) depicts severe narrowing of the intrahepatic vena cava inferior (arrowheads).

**Figure 5:**
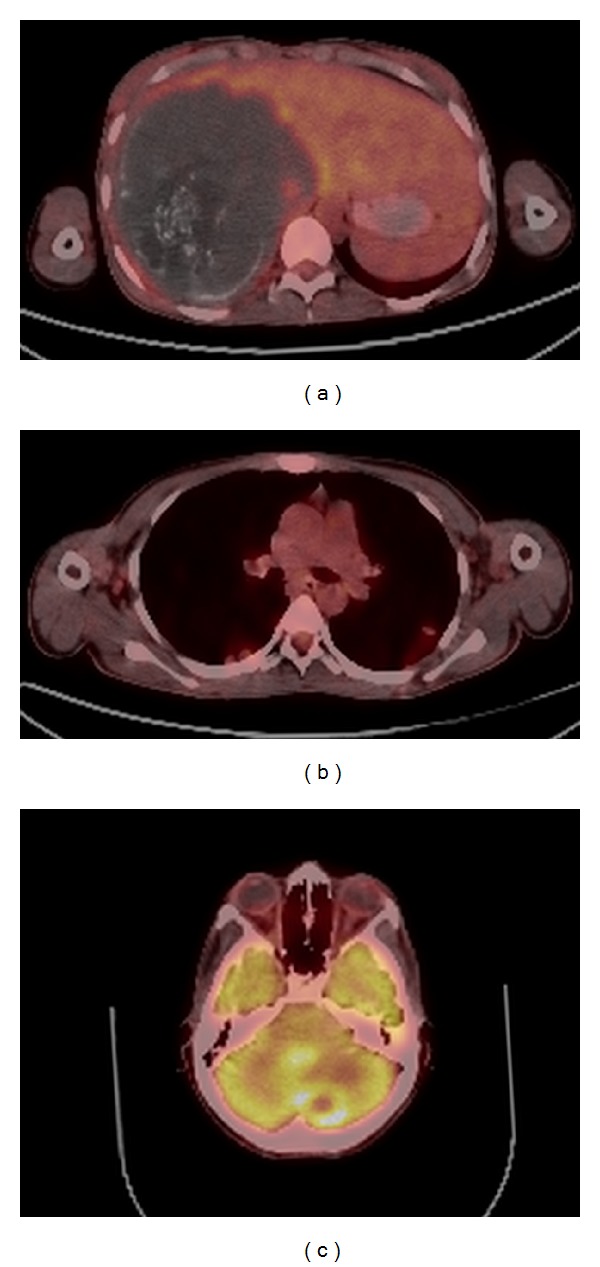
Whole-body PET-CT scan of the second case. AE lesion of the liver with only perilesional ^18^F-FDG uptake was seen on fused image (a). The evaluation of pulmonary nodules with PET-CT showed minimally increased metabolic activity (b). An axial slice from cerebellum revealed focal increased FDG uptake in the peripheral zone of the lesion (c).

**Figure 6:**
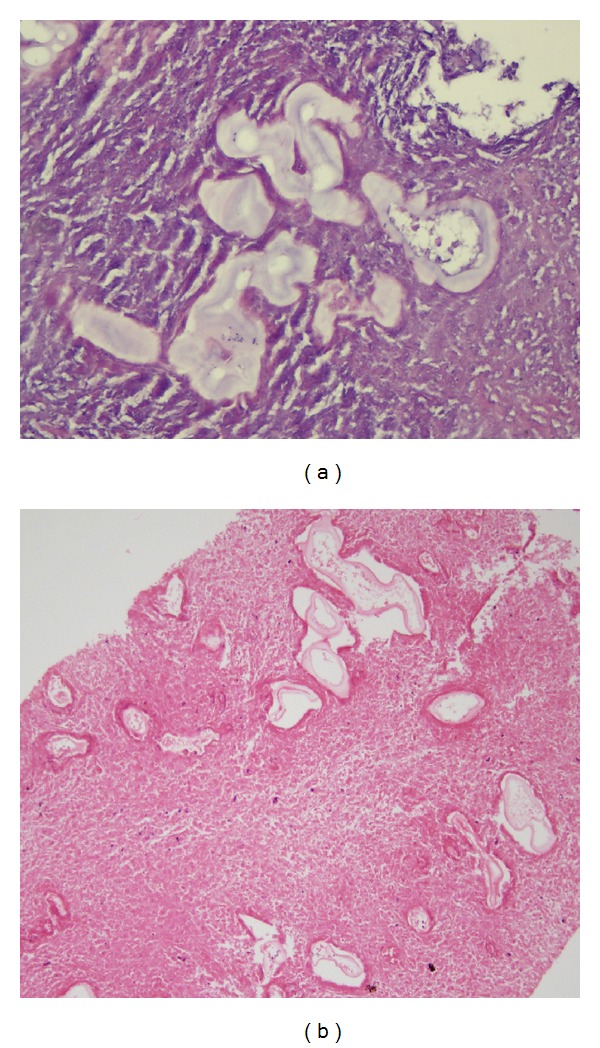
Pathologic examination revealed the germinative membranous structures of* Echinococcus multilocularis* in the liver parenchyma (400x magnification, hematoxylin and eosin staining) (a). Laminated membrane fragments are seen within the necrotic liver parenchyma of second case (20x magnification, hematoxylin and eosin staining) (b).

The patient developed abdominal pain and swelling during clinical follow-up, and contrast enhanced thoracoabdominal CT showed multiple solid lesions in both lungs (Figure 3(b)). Right adrenal gland enlargement with extensive low attenuation areas is noted on CT. Shape and contour of the right adrenal gland is preserved (Figure 3(c)). In the liver, CT showed a 16 × 15 cm hypodense mass lesion containing cystic-necrotic areas and multiple coarse calcified foci and showing mild peripheral contrast enhancement (Figures 4(a) and 4(b)). The lesion was causing thrombosis of the right branch of the portal vein and completely surrounding and severely constricting the intrahepatic segment of the inferior vena cava (Figures 4(c) and 4(d)). Additionally, widespread systemic collateral pathways were observed in the abdomen. The patient underwent whole-body ^18^F-FDG positron emission tomography-computed tomography (PET-CT) scan (Figure 5). As the imaging features strongly suggested metastatic malignancy, core needle biopsy of the mass lesion in the liver and surgical excision of the cerebellar lesion were performed. The histopathologic results were consistent with alveolar hydatid disease (Figure 6(b)). Liver transplantation was not possible for both patients because of the disseminated disease. No significant changes were observed after medical treatment with albendazole for six months.

## 3. Discussion

Alveolar echinococcosis is a disease with poor prognosis and may be fatal if left untreated [1, 2]. Symptoms vary depending on the involved organ and degree of involvement [2]. Initial findings and symptoms are nonspecific and may present as weight loss, abdominal pain, fever, jaundice, and hepatomegaly. In case of pulmonary involvement, chest pain, shortness of breath, cough, and mild hemoptysis may be seen [2, 3]. Cerebral involvement is rare and generally seen at the advanced stages of the disease. Symptoms related to increased intracranial pressure are epilepsy, hemiparesis, dysarthria, and cranial nerve palsies [2, 4].

Adrenal gland involvement in AE is very rare. Kamishima et al. [5] first reported it in a 77-year-old patient monitored for hypertension who was found to have primary right adrenal gland involvement. The multiple hypodense cystic areas and diffuse expansion that were observed inside the adrenal gland bear close resemblance to the CT findings of the right adrenal gland involvement seen in our second patient.

AE is diagnosed with a detailed history, physical examination, radiological imaging, and serological examinations [2]. While abdominal USG should be performed at initial examination, abdominal CT is the primary imaging modality in diagnosing AE, as it allows anatomical and morphological evaluation of lesions and observation of typical calcifications [2, 6]. Abdominal MRI, which has the highest sensitivity in demonstrating the components of parasitic lesions, must be part of preoperative evaluation along with MRCP, as they allow visualizing the neighboring organs, vascular structures, and biliary tracts [2, 6]. There are very few studies about diffusion weighted imaging (DWI) of AE lesions. Becce et al. [7] reveal that DWI is helpful for differentiation of hepatic AE lesions from other liver masses. According to this study, our two cases were relevant with type 3 lesions which include calcification and solid and necrotic components. The mean ADC_total_ for type 3 lesions were 1.73 ± 0.41 × 10^−3^ mm^2^/s. Our ADC-values were consistent with Becce et al. [7].

As seen in both of our patients, contrast enhancement is not seen in liver lesions except the minimal enhancement in the peripheral zones. In the second patient, abdominal CT angiography clearly demonstrated the extensive collateral veins that had formed between the left renal and hemiazygos veins as the result of inferior vena cava constriction and also showed the right portal vein branch thrombosis.

In case of brain involvement, cranial CT or MRI may reveal AE lesions [2–4]. MRI is particularly effective when demonstrating and following up intracranial lesions in AE [2]. Multilocular AE lesions generally appear heterogeneous hypointense on T2A and FLAIR images, with edema in the neighboring brain parenchyma. Postcontrast T1A images show peripheral contrast enhancement due to inflammatory reactive tissue surrounding the lesion [2]. In case of pulmonary involvement, imaging shows hypodense nodules with irregular shapes and minuscule calcified foci inside [2, 3]. The rupturing of lesions into the bronchial tree may result in cavitation [3]. In our first patient, we observed two thick-walled cavitating lesions in both lungs.

AE can be easily confused with primary hepatic malignancies and metastases and is misdiagnosed more often as compared to other helminth diseases [2, 3]. While imaging findings in AE are generally strongly suggestive of aggressive malignancy, the general condition of patients is uncharacteristically good. Our patients were residents of rural areas in Eastern Anatolia, where the disease is more prevalent. Metastatic malignancy was considered in differential diagnosis in both cases due to multiple organ involvement and aggressive appearance of lesions. Both patients underwent core needle biopsy of the liver lesions for definite diagnosis, and the histopathological findings were consistent with AE. There are some potential risks for suspected alveolar hydatid disease such as anaphylaxis and abdominal seeding after intervention. Intravenous access was established to avoid these situations. Also, epinephrine, prednisone, and antihistaminic drugs were prepared prior to biopsy. Fortunately, there were no complications after biopsies.

Recently, ^18^F-FDG PET-CT has been considered as a reliable method for differentiation between inactive and active AE lesions [8]. It has especially been proposed for the medical treatment planning of alveolar hydatid disease [8, 9]. In hepatic lesions, the preferred treatment method is partial hepatectomy [4]. Patients with unresectable lesions may benefit from liver transplantation [4]. Perioperative and long-term albendazole treatment has been reported to increase 10-year survival chances up to 80% [1]. It is essential to diagnose AE early in order to prevent a high number of unresectable lesions and the need for radical surgery [1].

In conclusion, radiologic imaging findings may contribute to the diagnosis of alveolar hydatid disease. AE lesions may be misdiagnosed as malignancies, and it is important for radiologists to have good command of typical imaging features of AE and to provide early diagnosis for patients from high prevalence areas, thereby guiding clinicians in the correct direction.
